# Supplementary material for: Loss of Y in regulatory T lymphocytes in the tumor micro-environment of primary colorectal cancers and liver metastases
Source: Sci Rep. 2024 Apr 24;14:9458. doi: 10.1038/s41598-024-60049-y (PMC11043399; doi:10.1038/s41598-024-60049-y)
Supplement: Supplementary file 1 — Supplementary Information. [file 41598_2024_60049_MOESM1_ESM.docx]

**Table S1. Basic clinical characteristics of CRC, LM_CRC and Ctrl patients qualified for the dissociation and FACS experiments.** Diagnosis: CRC – colorectal cancer, LM_CRC – liver metastasis of CRC, Ctrl – control patients.

| Diagnosis | No. of  subjects | Inclusion criteria | Exclusion criteria | Average age  (age range) | Type of  material  collected |
| --- | --- | --- | --- | --- | --- |
| CRC | 10 | Sex = male,  Primary tumor size  ≥ 3 cm | Neoadjuvant  therapy | 62  (42 – 74) | Tissues |
| CRC | 49 | Sex = male | N/A | 67  (30 – 94) | Blood |
| LM_CRC | 10 | Sex = male,  Primary tumor = CRC | N/A | 65  (42 – 78) | Tissues |
| LM_CRC | 13 | Sex = male,  Primary tumor = CRC | N/A | 65  (42 – 77) | Blood |
| Ctrl | 19 | Sex = male,  Age ≥ 65 | Cancer/Alzheimer  history | 68  (65 – 74) | Blood |

N/A – not applicable

**Table S2. CD4+ T cells and CTLs categorization of the scRNA-seq dataset derived from Zhang et al., based on patients, tissue type and cell subpopulations (separate Excel file)**

**Table S3. The list of top-ranked genes correlated with the expression of *TIGIT* in Tregs located in TME of CRC patients.**

| Gene | Protein | Correlation | Adjusted p-value |
| --- | --- | --- | --- |
| IL32 | Interleukin 32 | 0.31 | 1.82^-9^ |
| CD27 | CD27 | 0.30 | 7.92^-9^ |
| MAGEH1 | MAGE family member H1 | 0.25 | 7.23^-6^ |
| LAYN | Laylin | 0.25 | 1.14^-5^ |

**Table S4.** **The immune profile of an Atlas of cancer cells dataset derived from Nieto et al., distinguished based on patients, cell subpopulations and cancer types (separate Excel file)**


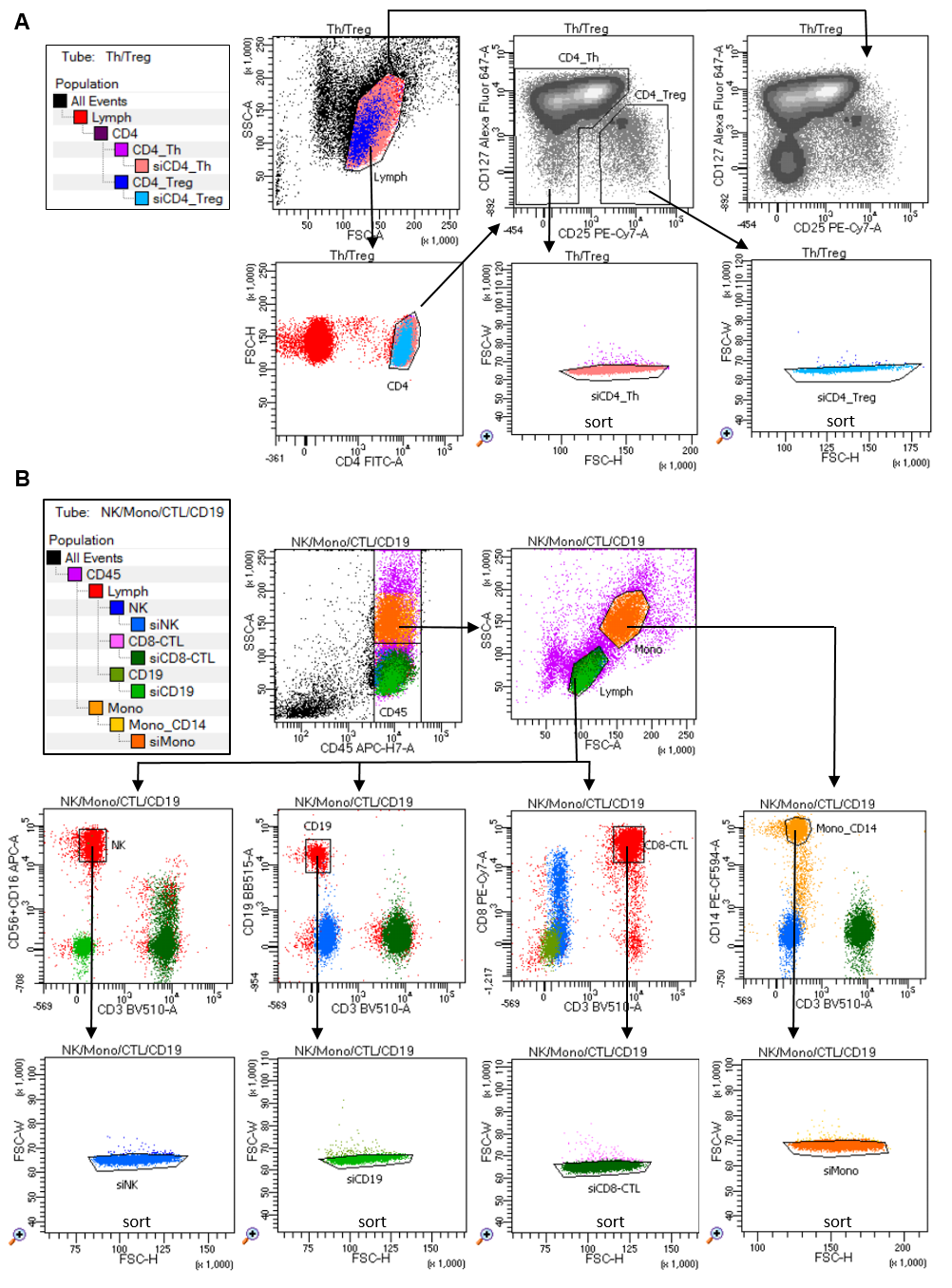


**
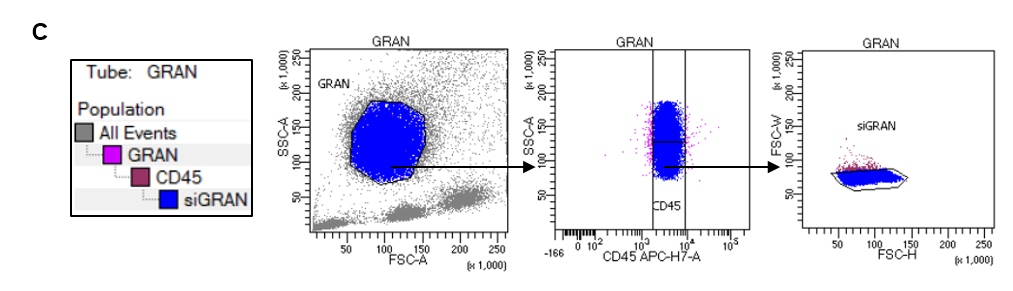
**

**Figure S1. Gating strategy used for fluorescence activated cell sorting of selected populations of leukocytes from peripheral blood.**

**Panel A:** Gating strategy for sorting of CD4 positive T cells – Regulatory T lymphocytes (Treg) and Helper T lymphocytes (Th).

**Panel B:** Gating strategy for sorting of NK cells (NK), Monocytes (Mono_CD14), B cells (CD19) and Cytotoxic T lymphocytes (CD8-CTL).

**Panel C:** Gating strategy for sorting of Granulocytes (Gran).

**
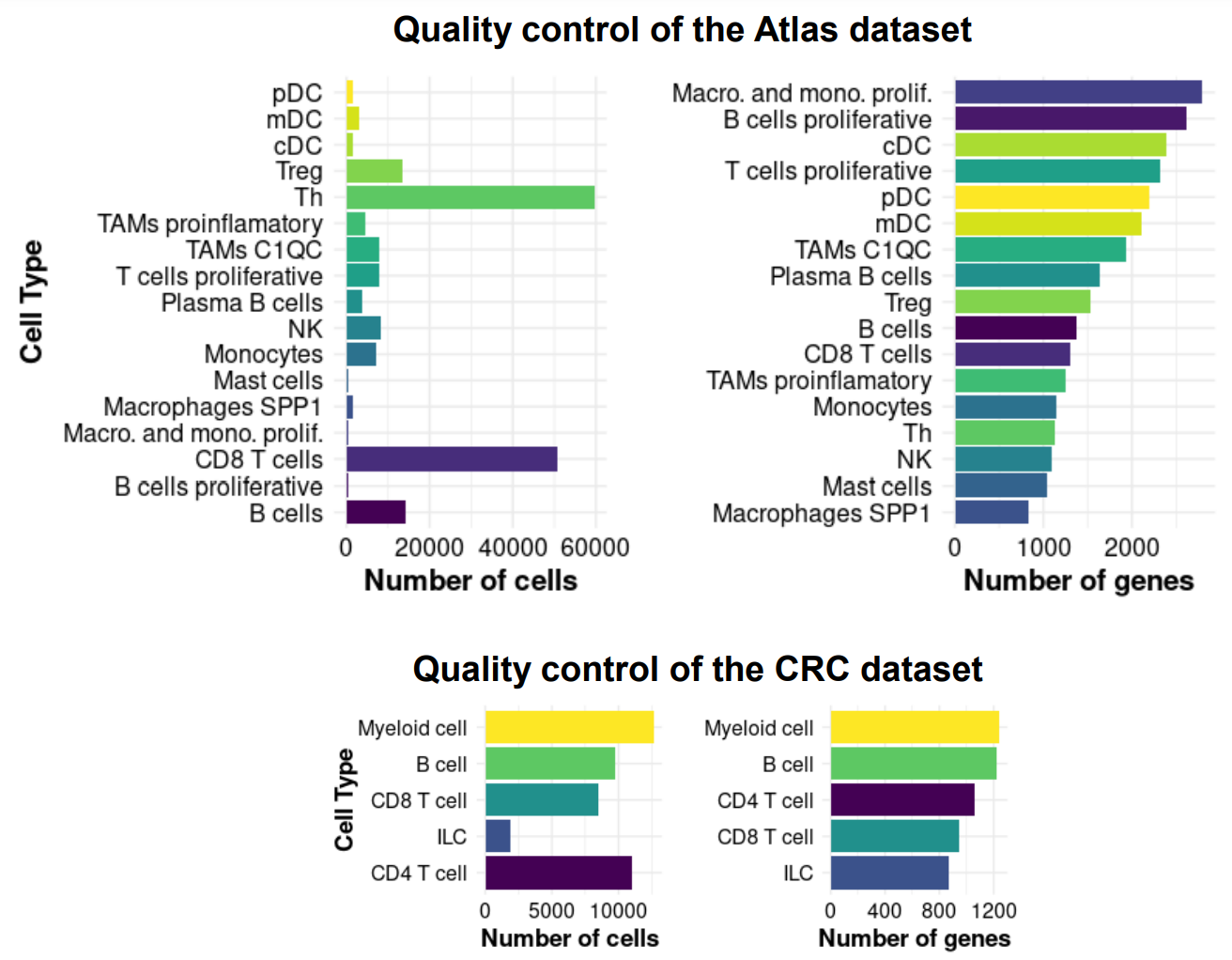
**

**Figure S2. Quality assessment of datasets.** The analysis of the Atlas dataset reveals over 50,000 CD8+ T cells (CTLs) and 70,000 CD4+ T cells (Th and Treg), indicating robust cell identification. Similarly, the CRC dataset exhibits approximately 10,000 cells for each CD8+ T and CD4+ T cell types, reflecting substantial cell numbers analyzed in our study. Moreover, the detection of more than 1,000 genes across all cell types in both datasets underscores the depth of sequencing achieved, affirming comprehensive transcriptome coverage.
